# Optical Methods in Fingerprint Imaging for Medical and Personality Applications

**DOI:** 10.3390/s17102418

**Published:** 2017-10-23

**Authors:** Chia-Nan Wang, Jing-Wein Wang, Ming-Hsun Lin, Yao-Lang Chang, Chia-Ming Kuo

**Affiliations:** 1 Industrial Engineering and Management, National Kaohsiung University of Applied Sciences, Kaohsiung 80778, Taiwan; chungys@fotech.edu.tw; 2 Institute of Photonics and Communications, National Kaohsiung University of Applied Sciences, Kaohsiung 80778, Taiwan

**Keywords:** personality traits, fingerprint classification, fingerprint types

## Abstract

Over the years, analysis and induction of personality traits has been a topic for individual subjective conjecture or speculation, rather than a focus of inductive scientific analysis. This study proposes a novel framework for analysis and induction of personality traits. First, 14 personality constructs based on the “Big Five” personality factors were developed. Next, a new fingerprint image algorithm was used for classification, and the fingerprints were classified into eight types. The relationship between personality traits and fingerprint type was derived from the results of the questionnaire survey. After comparison of pre-test and post-test results, this study determined the induction ability of personality traits from fingerprint type. Experimental results showed that the left/right thumbprint type of a majority of subjects was left loop/right loop and that the personalities of individuals with this fingerprint type were moderate with no significant differences in the 14 personality constructs.

## 1. Introduction

Understanding the personality traits of one’s self and others contributes to harmonious interpersonal relationships. However, getting to know one’s self and others in a short period of time is not an easy task, and inducing the personality traits of others is an even more difficult undertaking. In the Western world, studies on personality traits have a long and broad history [1]. Many related studies have since followed, but the number of proposed personality characteristics has remained high. It was for this reason that Cattell [2] converted these characteristics into 16 types of personality factor questionnaires. Later, Fiske [3] performed a follow-up verification of Cattell’s research and derived the “Big Five” personality dimensions. In 1963, Norman [4] verified Cattell’s procedures and announced that the five major factors constituted a reasonable method of personality classification.

Research on personality traits is core to many major disciplines, such as medicine, psychology and corporate management, whether for theoretical investigation or practical application [5]. Personality traits stem from a consistent behavioral model and internal processes within each individual, allowing the individual to identify with a consistent behavioral model in different situations. The internal processes of personality traits include emotions, motivation and cognition. Although these processes occur at a deep level, they influence human behavior and feelings [6]. Additionally, other studies have attempted to classify individuals into different personality types [7]. For hundreds of years, the Chinese people have used physiognomy, palmistry (the ridges on the skin of the palm), bone reading and other methods related to physiological features to divulge an individual’s personality traits and fortune. To date, however, there are no studies that support a relationship between personality traits and fingerprints, which are an individually unique physiological feature.

Two features of fingerprints that are particularly important: (1) fingerprints do not change with time; and (2) every individual’s fingerprints are unique [8]. Due to the above-described two characteristics, fingerprints have long been used for identification purposes [9,10]. Medina-Pérez proposed a new feature representation containing clockwise-arranged minutiae without a central minutia, a new similarity measure that shifted the triplets to find the best minutiae correspondence, and a global matching procedure that selected the alignment by maximizing the amount of global matching minutiae [11]. In comparison with six verification algorithms, the proposed method achieved the highest accuracy in the lowest matching time. Ballan and Gurgen [12] presented a method for fingerprint recognition based on principal component analysis and point patterns (minutiae) obtained from the directional histograms of a fingerprint. This study gave the same performance as that of the uncompressed data, but reduced computation. Yang et al. used fusion to enhance the biometric performance in template-protected biometric systems [13]. They investigated several scenarios (multi-sample, multi-instance, multi-sensor, multi-algorithm and their combinations) on the binary decision level and evaluated the performance and fusion efficiency on a multi-sensor fingerprint database with 71,994 samples. Fingerprint image quality improvement was proposed in [14]. The algorithm consists of two stages. The first stage is decomposing the input fingerprint image into four sub-bands by applying the two -dimensional discrete wavelet transform. At the second stage, the compensated image is produced by adaptively obtaining the compensation coefficient for each sub-band based on the referenced Gaussian template. The method concluded an improved clarity, quality and continuity of ridge structures, and therefore, the accuracy is also increased. Background and the blurred region of fingerprint images are also removed. Bartunek et al. [15] presented several improvements to an adaptive fingerprint enhancement method that is based on contextual filtering. Based on the global analysis and the matched filtering blocks, different forms of order statistical filters were applied. These processing blocks yield an improved and adaptive fingerprint image processing method. Yang et al. [16] proposed a novel and effective two-stage enhancement scheme in both the spatial domain and the frequency domain by learning from the underlying images. They first enhanced the fingerprint image in the spatial domain with a spatial ridge-compensation filter by learning from the images. With the help of the first step, the second stage filter, i.e., a frequency band-pass filter that was separable in the radial and angular frequency domains was employed. The experimental result showed that their algorithm is able to handle various input image contexts and achieves better results compared with some state-of-the-art algorithms over public databases and is able to improve the performances of fingerprint-authentication systems.

Fingerprints are closely related to genetics [17]; however, in the fields of biostatics and psychology, there are currently no studies indicating any relationship between fingerprints and personality traits. Therefore, using fingerprints to induce personality traits is an undeveloped area in scientific research. If the corresponding relationship between fingerprints and personality traits could be determined, this would be an important contribution to science. Since personality traits have a certain degree of stability, continuity and uniqueness and the left/right hand fingerprints of each person are unique, the relationship between these two features is a worthwhile topic for in-depth research. The Big Five personalities have generated substantial interest among personality researchers [3]. The Big Five is a model based on common language descriptors of personality. When factor analysis (a statistical technique) is applied to personality survey data, some words used to describe aspects of personality are often applied to the same person. These five factors are openness to experience (inventive/curious), conscientiousness (efficient/organized), extraversion (outgoing/energetic), agreeableness (friendly/compassionate) and neuroticism (sensitive/nervous). 

The purpose in this study is to evaluate the generalizability of Big Five personality factor inventories as inducers of a common set of criteria, criteria representing classes of left and right thumb fingerprints. By assessing people using multiple criterion variables to measure the Big Five personality constructs, the same measure results normally will have the same personality constructs. If the Big Five inventories are all designed to measure equivalent dimensions of personality, then they should show a nontrivial amount of agreement in the variables they are able to induce. Constructing valid measures of personality variables should induce fingerprint classes, assuming those classes have personality determinants. This is especially true of Big Five inventories because those factors are presumed to account for most of the personality-based variation in fingerprints.

This study used classification technology to derive eight fingerprint types and combined these with questionnaire survey results to construct a new “System for Induction of Personality Traits from Fingerprints”. Following the research of Costa and McCare [18], this study also summarized 14 personality constructs with Eigen values greater than one from the “Big Five” personality factors. We performed a principal components analysis of the data and found 14 components with Eigen values larger than one. Then, we created 14 scales each comprised of one of the 14 groups of items indicating the 14 components with Eigen values larger than one. The prototype of this system was modified and completed based on the fingerprints and questionnaire feedback of 362 test subjects. This study recruited a separate group of 351 subjects for the live testing of the system. The experimental results showed that the thumbprint types of the left and right hands were correlated with personality traits. Subjects in the left loop/right loop fingerprint category accounted for the largest group (41.8%). The second largest group was the S-type/S-type (twin loop/twin loop) type (13.5%), followed by the eddy/eddy type (12.1%). The personality traits of the latter two groups showed significant differences in some constructs. 

Whilst better known in medication, double blind experiments are adopted in this paper. Surveys with questionnaires are used to keep credibility so the chance of observer’s bias can be minimized. The framework of the following sections in this paper is as follows: Section 2: research framework and flow figure, expansion of the “Big Five” personality factors into 14 constructs, design of the personality traits questionnaire and the “System for Induction of Personality Traits from Fingerprints”; Section 3: statistical analysis and post-test verification of the survey results on the relationship between personality traits and finger classification; Section 4: conclusions.

## 2. Research Methods

### 2.1. Research Framework

**Figure 1:**
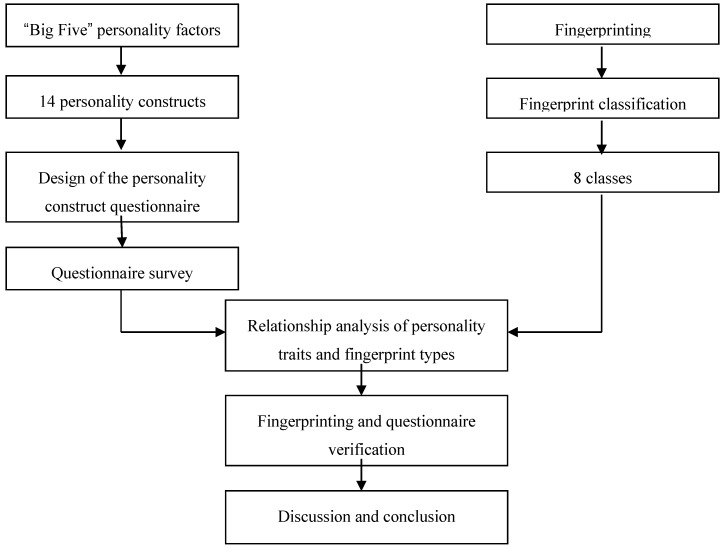
Research framework and flowchart.

The “Big Five” personality factors are advanced global factors that describe human personality, and the 16 personality factors are basic primary factors. This study summarized the questionnaire results after analysis of both global and primary factors. From these data, 14 personality constructs appropriate for describing the personalities of the test subjects were designed. A questionnaire was designed based on the 14 personality constructs. Along with the implementation of the questionnaire survey, an optical fingerprint machine of SecuGen Hamster Plus [19] was used to capture the left/right thumbprints of the test subjects. The fingerprint sensor features smart capture technology and switches on the scanner whenever it detects a finger. Thumb samples of both hands are collected, where 282 participants are mostly university students, and their ages are within the interval from 18 to 50 years old. The biometric data were collected in different periods within 3 months. A new fingerprint classification algorithm was used for fingerprint categorization. After relationship analysis of fingerprint types and the 14 personality constructs, this study compiled a table of associations between the eight fingerprint types and the 14 personality constructs. To verify the accuracy of the association table, this study performed post-testing with different subjects. After the questionnaire survey and fingerprinting had been re-conducted, this study modified the content of the association table. The conclusions of this study were formed after discussion of the pretest and posttest results. Figure 1 illustrates the framework and flow of this research. 

### 2.2. The 14 Constructs of Personality

The “Big Five” personality factors, which have been analyzed and verified by Norman [4], Goldberg [20] and McCrae and Costa [21], are as follows: neuroticism, extraversion, openness, agreeableness and conscientiousness. This study referred to the Big Five personality factors and the 16 personality factors, summarized the questionnaire results according to the personality scores of test subjects and designed a personality trait questionnaire composed of the 14 personality constructs. Neuroticism is the tendency of an individual to experience anxiety or nervousness. This study sub-divided neuroticism into sentimentalism, impulsiveness and strong self-esteem. Extraversion refers to the characteristics and strength of an individual in interpersonal interaction. This study sub-divided extraversion into activeness and passivity. Openness refers to the degree of risk that an individual can accept with regard to new things. This study sub-divided openness into enthusiastic attitude and good money concept. Agreeableness refers to the cognition, affection and attitude displayed by an individual toward various situations or matters. This study sub-divided agreeableness into socially harmonious methods of operation, concern for others’ well-being and impatience. Conscientiousness refers to the determination and self-discipline of an individual. This study sub-divided conscientiousness into strong sense of responsibility, slow method of operation, focused attention and strong leadership ability. Table 1 describes the 14 personality constructs in detail.

### 2.3. Fingerprint Classification

**Figure 2:**
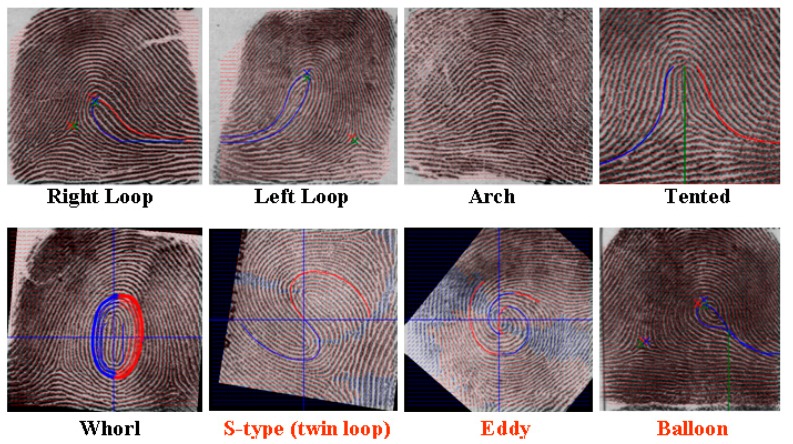
The eight fingerprint types.

The actual number of different types of human fingerprints is currently unknown; however, a majority of studies use the five main categories proposed by Henry [22]: Right loop, left loop, tented arch, arch and whorl. This study used an optical fingerprinting machine to capture original images of fingerprints. These original images are often accompanied by deformation caused by problems such as dry, wet, damaged or scarred fingerprints and uneven application of force by the fingerprinting machine when capturing the image. Therefore, enhancement of the images is essential [23]. Fingerprint classification is a coarse level method of partitioning a fingerprint database into smaller subsets, which reduces the search space of a large database. To determine the class of the query fingerprint, only search templates with the same class as the query were used. Inputs are the fingerprint impressions from right and left thumb fingers of an individual. If the size of the database is *n* and *c* is the number of classes, the search space without classification is $n^{2}$. With fingerprint classification, the search space with classification is *n*/*c*. We made an extensive study of the occurrence of fingerprints and indexed them into eight major classes as shown in Figure 2.

After using histogram specification and ridge/valley energy analysis, this study performed energy image projection analysis in eight different directions at an angle of 45° to capture fingerprint regions of interest (ROI). Through the above-described methods, this study classified fingerprint into four categories. They are whorl (plain whorl), S-type (double-loop whorl), eddy (accidental whorl) and balloon (central pocket loop whorl). To accurately trace flow lines to determine fingerprint type, this study used the Poincare index-based modified hierarchical singularity detection algorithm after orientation field estimation to detect the location of singularity. The three-stage pyramid singularity detection algorithm designed by this study can accurately locate the point of singularity through progressively narrowing the detection range. Lastly, after initial type selection had been performed based on the number and type (delta, core point) of the singular points, fingerprint classification was conducted by tracing the flow of the orientation field surrounding the point of singularity and establishing related rules of judgement. This study is interested in finding the exact location of the core point defined by the Henry system and therefore traces the skeletonized ridge curves with 8-adjacency to explore wavelet extrema at one-pixel increments by starting at 10 pixels apart from two sides. The highest extrema in the ridge curve corresponds to the candidate of the core point. We devise two 8-adjacency grids to locate the wavelet extrema. Beginning from two opposite ends and moving toward the center of the sub-region, the black-color pixel of each grid is designated as the central point to trace. Based on this central point, the moving guideline is as follows: if the gray-level of the adjacent pixel is 0, then move toward that pixel, where the number shown in the grid indicates the moving sequence. This method makes it possible to follow the real track of the ridge curve. Whenever a singularity is detected, its location is noted. It is common to have multiple findings of the core point candidate with small vertical displacements, and the area underneath the lowest ridge curve is circumscribed for locating the core point. In the Henry system, the exact core point location can be performed as follows: (a) locate the topmost extrema in the innermost ridge curve, if there is no rod; (b) otherwise, locate the top of the rods. The final eight categories derived are as follows (see Figure 2): right loop, left loop, arch, tented arch, whorl (plain whorl), S-type (double-loop whorl), eddy (accidental whorl) and balloon (central pocket loop whorl).

### 2.4. Questionnaire Design and Survey

This study designed a questionnaire with 74 closed questions since there is no measure of personality and fingerprint available. Since the 74 closed questions are many, this study does not provide all question items in this study. The 74 questions followed the 14 different personality constructs [18]. Each construct had at least 3 questions prepared by this study to confirm the results of respondents. However, this study also designed the reliability analysis and validation analysis for verifying the questionnaire design and post-test verification for final result checking. Based on reliability analysis and validation analysis, we confirmed that our questionnaire has the right dimensionality and composition. After factor analysis, the recovered questionnaire data were used to develop 14 personality constructs. The information collected from the questionnaire, along with the left/right thumbprints taken from the test subjects, was used in conjunction with the personality constructs to derive the relationship between fingerprint type and personality traits. A 5-point Likert scale was used for the personality trait questionnaire; 1–5 points were respectively assigned to the options of strongly disagree, disagree, neither agree, nor disagree, agree and strongly agree.

Sampling in this study was conducted via the following steps:

1. Research targets: The use of fingerprints involves personal privacy issues, and the agreement of the respondent with regard to using his/her fingerprints for research is difficult to obtain. Thus, for the sake of convenience in collecting information, this study used non-probability proportional sampling methods and selected the National Kaohsiung University of Applied Sciences at Taiwan as the site for questionnaire distribution. The main respondent targets were students in the Department of Industrial Management, Continuing Education Division.
2. Questionnaire response process: The process of filling out the questionnaires proceeded according to classes (as units) and was arranged according to students’ class hours. After the left/right thumbprints of the respondents had been collected into the fingerprint classification system, the questionnaires were filled out.
3. Results: This study distributed 362 questionnaires. After the questionnaires had been collected and any invalid questionnaires removed, the number of valid questionnaires was 282, resulting in a valid recovery rate of 75.4%. After the average value of the questions in each construct had been processed, these data were used for the final score of each respondent.

### 2.5. Statistical Analysis and Testing

Reliability analysis: This study used Cronbach’s α [24] to measure the reliability of the questionnaire. According to the research of DeVellis [25], a reliability coefficient value of 0.7 and up is acceptable. The overall Cronbach’s α value for the 74 items in this questionnaire was 0.799, indicating that this questionnaire had high reliability. The Cronbach’s α value for the 14 constructs also exceeded 0.7, indicating the reliability of the data. The Cronbach’s α value of individual constructs of sentimentalism, impulsiveness, strong self-esteem, liveliness, passivity, positive attitude, good money concept, socially harmonious method of operation, concern for others’ well-being, impatience, strong sense of responsibility, slow method of operation, focused attention and strong leadership ability are 0.797, 0.786, 0.793, 0.771, 0.829, 0.770, 0.792, 0.783, 0.764, 0.795, 0.776, 0.808, 0.804 and 0.771, respectively.

Validity analysis: Validity is the degree to which the questionnaire accurately measures what it is intended to measure; in other words, the degree to which it reaches the goals of measurement. This study used factor analysis to obtain the total variance explained by the questionnaire, and this value was used to measure validity. However, Sharma [26] advised against relying solely on the results of Bartlett’s test of sphericity to determine whether data are suitable for factor analysis, because the validity of Bartlett’s test of sphericity is easily influenced by sample size. Therefore, this study mainly used the Kaiser–Meyer–Olkin (KMO) measure and Bartlett’s test of sphericity to determine whether the data were suitable for factor analysis. The KMO coefficient was used to measure whether each variable had sampling adequacy. A KMO coefficient of 0.9 and up was considered upper level, 0.8–0.89 was considered moderate level and lower than 0.5 was an unacceptable level. The KMO and Barlett test results show that KMO = 0.536, indicating that the data in this study were in an acceptable range with regard to sampling adequacy. Bartlett’s test of sphericity also reached a level of significance (*p* < 0.001), indicating that the data were suitable for factor analysis. The number of factors in factor analysis could have been determined by the relationship of the 74 questionnaire items. This study used principal component analysis for repeated estimation until the estimation of commonalities converged. Varimax was then used for rotation. The analysis results showed that the Eigen value of 14 questions exceeded 1, and the total explainable variance was 74.75%, surpassing the minimum requirement of 50%. Therefore, this study used these 14 factors as personality constructs. 

## 3. Experimental Results

**Figure 3:**
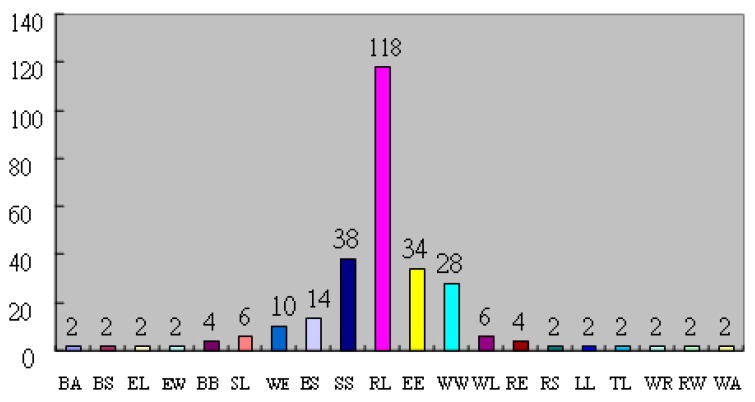
Fingerprint type statistics. BA: Balloon/Arch; BS: Balloon/S-type; EL: Eddy Loop/Left Loop; EW: Eddy Loop/Whorl; BB: Balloon/Balloon; SL: S-type/Left Loop; WE: Whorl/Eddy Loop; ES: Eddy Loop/S-type; SS: S-type/S-type; RL: Right Loop/Left Loop; EE: Eddy Loop/Eddy Loop; WW: Whorl/Whorl; WL: Whorl/Left Loop; RE: Right Loop/Eddy Loop; RS: Right Loop/S-type; LL: Left Loop/Left Loop; TL: Tent/Left Loop; WR: Whorl/Right Loop; RW: Right Loop/Whorl; WA: Whorl/Arch.

This study recovered 282 valid questionnaires. Initial results indicated that in left/right hand fingerprint types, the right hand fingerprints did not show arch type; arch type was also not found in some of the left hand fingerprints. Among the fingerprint types, the left loop/right loop type accounted for the largest group of test subjects (118 subjects), followed by the S-type/S-type (38 subjects), eddy/eddy type (34 subjects) and whorl/whorl type (28 subjects). The summary of type numbers is shown in Table 2 and Figure 3. In the questionnaire, 1–5 points each were assigned to the Likert scale options (strongly disagree, disagree, neither agree, nor disagree, agree, strongly agree). This study calculated the mean and standard deviation of each of the 14 personality constructs. Based on the responses on Likert items, this study derived the interrelationship between fingerprint type and personality construct; for details, please see Table 3. 

**Figure 4:**
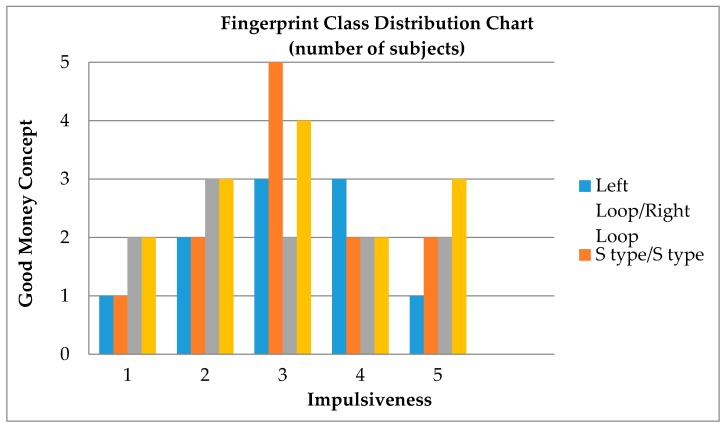
A sample distributed chart of personality traits.

Table 3 shows that S-type/right Loop had the highest overall average in the 14 constructs, indicating that subjects with this fingerprint type demonstrated significant inclination in personality traits. The overall average of arch/whorl was the second highest in the 14 constructs, particularly with regard to the traits of “socially harmonious method of operation”, “concern for others’ well-being”, “enthusiastic attitude” and “strong sense of responsibility”; the overall average in terms of these four constructs even exceeded that of S-type/right loop. This indicated that individuals with this fingerprint type have outstanding leadership qualities. Additionally, to summarize the distribution trend of fingerprint type and personality constructs, a sample distributed clustering image of fingerprint type and personality traits is shown in Figure 4, using four of the fingerprint types that accounted for a higher number of subjects and two personality traits. This is a sample distributed chart of personality traits based on fingerprint type, using four of the fingerprint types that accounted for a number of subjects and two personality traits (1–5 points each were assigned to the Likert scale options).

## 4. Discussions

This study used the statistical concept of interval estimation to determine the personality traits corresponding to different fingerprint types. Using the construct of “socially harmonious method of operation” as an example: this study first calculated the average ($\bar{Χ}=3.721$) and standard deviation (S = 0.6307) of this construct in the 282 questionnaires collected and then calculated the 95% confidence interval for the mean: 3.6169 ≤ µ ≤ 3.8215. The researchers then determined whether the average of the personality traits corresponding to each fingerprint type fell within the interval. The results of the interval estimation are shown in Table 3.

### 4.1. Post-Test Verification I

The purpose of verifying the questionnaire was to test the accuracy level of the system. Verification of the questionnaire consisted of three parts: The first part was a simple personality trait questionnaire consisting of 15 questions; the second part was fingerprinting; and the third part was the test subjects’ rating of the accuracy level of the system. The assessment options were extremely inaccurate to extremely accurate (1–10 points). The process of questionnaire verification was as follows: After test subjects had filled in the personality trait questionnaire, their left/right thumbprints were taken. Fingerprint classification was used to determine personality constructs. Test subjects then rated the accuracy level of the system according to their personality construct placement. This study distributed 56 verification questionnaires in total and recovered 45 valid questionnaires, making a recovery rate of 80.34%. Initial results showed that only 12 fingerprint types had been obtained, among which left loop/right loop accounted for the highest proportion. The number summary of fingerprint type is shown in Table 4. Table 4 shows that the left loop/right loop accounted for the highest proportion (14 subjects), followed by eddy/eddy (nine subjects), S-type/S-type (six subjects) and whorl/whorl (five subjects). This study used the concept of interval estimation on the fingerprint type data collected, to determine the relationship between fingerprint type and personality constructs, as shown in Table 5. 

Table 5 shows that some of the verification questionnaire results regarding personality traits that correspond to fingerprint types differ from the pre-test questionnaire results (Table 3). There are some discrepancies between pre-test and post-test. We amended the legend as the discrepancy between pre-test and post-test shown in the highlighted cell. Possible reasons for these differences are summarized below:

1. Insufficient sample number (subjects): Obtaining test subjects for the post-test verification was difficult, resulting in a small sample number. This may have caused some errors in the process of using interval estimation to determine corresponding personality traits, producing some differences in the results.
2. Insufficient number of samples for different fingerprint types: This study noticed that the number of samples for certain fingerprint types, such as whorl/right loop and left loop/S-type, was very few. The pre-test results and the verification results show that the number of samples obtained for some fingerprint types was very few; this insufficient sample number may have produced error in the process of summarizing results. By contrast, the amount of samples collected in the pre-test and verification processes for certain fingerprint types such as left loop/right loop, whorl/whorl and eddy/eddy, is significantly higher than others. In the verification process, the results of personality traits corresponding to these four fingerprint types were significantly more consistent as compared to other fingerprint types.

This study used the mean confidence interval to determine whether the differences between the pre-test results and those of post-test verification were significant. In view of the individual uniqueness of fingerprints, this study assumed that fingerprint types were mutually independent. Below is a simple explanation using the fingerprint type left loop/right loop and the construct “socially harmonious way of operation”:

Pre-test questionnaire: $n_{1}$ = 59, $\bar{X_{1}}=3.65$, $S_{1}$ = 0.665; verification questionnaire: $n_{2}$ = 14, $\bar{X_{2}}=3.44$, $S_{2}$ = 0.8644; these data were used for a mean difference test (α = 0.05). The resulting mean difference confidence interval was [−0.2736, 0.6936], and the 95% confidence interval included zero; therefore, we can assume that there is no significant difference between the pre-test and post-test results for the fingerprint type left loop/right loop and the construct “socially harmonious method of operation”.

### 4.2. Post-Test Verification II

This study randomly sampled interested participants as subjects for this test. Following the test, participants filled out an accuracy questionnaire on their degree of satisfaction with using fingerprint types to analyze personality traits. There were 306 participants in this test. With the inclusion of the 45 valid questionnaires recovered from Post-Test Verification I, this totals to 351 results for the accuracy survey. The average was 7.1268, and the mode was eight. This means that subjects rated the accuracy of the system developed by this study at more than 70%. The results of the accuracy survey are shown in Table 6. This study used the above data for hypothesis testing. Because researchers wished to determine whether the outcome significantly exceeded the median (5), the null hypothesis was H0: μ ≦ 6, and the alternative hypothesis was H1: μ > 6. The test result was 9.7. At the α = 0.001 level of significance, the mean was shown to significantly exceed six. Therefore, we can infer that the results obtained from this system were very accurate.

## 5. Conclusions

Analysis and accurate induction of personality traits is extremely valuable in both daily life and academic research. This study designed 14 personality constructs and implemented the questionnaire survey and fingerprinting through innovative fingerprint classification technology. Through comparison of pre-test and post-test results, this study realized the induction ability of personality traits from fingerprint type. Detailed conclusions are as follows:

1. Validity and reliability analysis showed that personality traits and fingerprint type are statistically correlated. Additionally, more than 70% of subjects were satisfied with the accuracy of the results of personality trait induction.
2. The results of the relationship between personality trait and fingerprint type showed that subjects with the left loop/right loop fingerprint type accounted for the largest proportion and were more moderate in terms of personality traits. In other words, subjects with this fingerprint type did not exhibit any especially prominent personality trait in the 14 constructs. The overall average of S-type/left loop was the highest among the 14 personality constructs, indicating that subjects with this fingerprint type had generally obvious personality traits. Arch/whorl had the second highest overall average in the 14 personality constructs, particularly in the constructs of “socially harmonious method of operation” (5.00), “strong sense of responsibility” (4.83), “enthusiastic attitude” (4.50) and “concern for others’ well-being” (4.11). In these four constructs, the overall average of arch/whorl exceeded that of S-type/right loop, indicating that subjects with this fingerprint type had strong leadership qualities.
3. Among the 20 left/right fingerprint types derived from fingerprint classification, four fingerprint types accounted for a majority of subjects. The type accounting for the highest proportion was left loop/right loop (pre-test: 42%, Post-Test Verification I: 34%), followed by eddy/eddy (pre-test: 14.29%, Post-Test Verification I: 12%); S-type/S-type (pre-test 13.74%, Post-Test Verification I: 13%) and whorl/whorl (pre-test: 10.44%, Post-Test Verification I: 10%).
4. In the process of investigating fingerprint type, this study found an additional three fingerprint types apart from the five known types: S-type, eddy and balloon. This is a new discovery in fingerprint classification. With regard to accuracy, the classification accuracy of the eight fingerprint types reached 89.76%.

Research on personality traits, whether in terms of theoretical or practical application, is a key topic in modern research domains. Accurate induction of personality traits is a field of human research that not only urgently requires development, but also offers high practical value in such circumstances as schools selecting suitable students or corporations recruiting suitable personnel. Fingerprints are a unique human biological characteristic. This study is the first to propose a method of using fingerprint type to induce personality traits, as well as to verify the effectiveness of this method. Future research can build on the results of this study and expand research on fingerprint type to other areas, such as the relationship between fingerprint type and learning ability or the industries to which individuals with different fingerprint types are more suited. Moreover, more samples need to be prepared to study in this field to verify the original results and discover new findings.

## Figures and Tables

**Table 1:** Fourteen constructs of personality.

14 Constructs of Personality	Description of Constructs
Sentimentalism	Emotionally sensitive and easily becomes sentimental; emotionally vulnerable to external stimuli and reveals true feelings.
Impulsiveness	The link that precedes the conversion of one’s feelings, perception and thoughts into actions: the desire before the action. The word ‘rash’ is commonly used to describe such actions that were not previously thought out.
Strong self-esteem	Maintains self-respect and dignity; does not allow discrimination, stigmatization or attack from others.
Liveliness	Refers to the *qi* (energy flow or vitalism) exhibited by an individual; lively people drive the surrounding atmosphere, influence people around them and attract more attention in a group.
Passivity	Always keeps personal opinions and decisions to one’s self; does not take the initiative to directly express one’s self, but is always waiting for the other person to ask.
Positive attitude	Enthusiasm is a type of outward manifestation of desire; whether the job is actually well done is considered secondary. It is a type of zeal, an attitude of full immersion without distraction.
Good money concept	Ensures that expenditure matches income and avoids debt; considers one’s status and position when spending money to select products that are appropriate for one’s self.
Socially harmonious method of operation	Behaves appropriately, does not openly offend others, is concerned that matters reach a socially harmonious and satisfactory conclusion.
Concern for others’ well-being	Puts one’s self in others’ shoes and considers others in any situation; an attitude of “not doing unto others what you would not want done unto yourself”.
Impatience	Unsettled and irritable behavior when facing situations that require waiting or delay.
Strong sense of responsibility	Fulfills one’s obligations regarding any matter; is always aware of possible consequences no matter how great or small the matter and is able to assume one’s proper responsibility.
Slow method of operation	Is slow and calm in any situation; gives others the impression of a slow and unconcerned attitude.
Focused attention	Is not easily influenced by the external environment when working or engaging in various matters.
Strong leadership ability	Plays the role of a leader in groups; is good at organizing/assigning tasks and coordinating interpersonal relationships; provides a team with sufficient centripetal force.

**Table 2:** Fingerprint type number summary.

Fingerprint Type	Left Loop	Right Loop	Tent	Arch	Whorl	Eddy Loop	S-type	Balloon
Left Loop	2	118	2	0	6	2	6	0
Right Loop	0	0	0	0	2	0	0	0
Tent	0	0	0	0	0	0	0	0
Arch	0	0	0	0	2	0	0	2
Whorl	0	2	0	0	28	2	0	0
Eddy loop	0	4	0	0	10	34	0	0
S-type	0	2	0	0	0	14	38	2
Balloon	0	0	0	0	0	0	0	4

**Table 3:** Fingerprint types and personality constructs.

Fingerprint Type/Personality	Socially Harmonious Method of Operation	Sentimentalism	Liveliness	Concern for Others’ Well-Being	Strong Self-Esteem	Impatience	Impulsiveness	Positive Attitude	Strong Sense of Responsibility	Slow Method of Operation	Passivity	Focused Attention	Strong Leadership Ability	Good Money Concept
Left Loop/Right Loop	△	△	△	△	△	△	△	△	△	△	△	△	△	△
Whorl/Right Loop	Χ	Χ	○	△	△	△	Χ	Χ	△	Χ	△	Χ	Χ	○
Eddy/Right Loop	Χ	○	Χ	△	○	○	△	Χ	Χ	○	○	△	○	○
S-type/Right Loop	○	○	△	○	○	○	○	○	○	○	○	○	○	○
Left Loop/Left Loop	○	△	Χ	△	○	Χ	Χ	○	○	Χ	○	Χ	Χ	Χ
Left Loop/Tent	Χ	Χ	○	△	○	△	○	Χ	Χ	○	○	Χ	Χ	○
Right Loop/Whorl	Χ	○	Χ	Χ	△	△	○	Χ	Χ	○	○	Χ	Χ	○
Left Loop/Whorl	○	△	△	○	△	△	Χ	○	△	○	△	Χ	○	Χ
Arch/Whorl	○	△	○	○	○	○	Χ	○	○	Χ	Χ	○	○	Χ
Whorl/Whorl	△	○	△	△	Χ	△	○	○	△	△	Χ	Χ	△	○
Eddy/Whorl	○	△	Χ	△	○	△	△	○	○	○	Χ	○	○	△
Left Loop/Eddy	△	△	○	○	○	△	○	△	Χ	Χ	Χ	Χ	○	Χ
Whorl/Eddy	Χ	○	Χ	△	○	○	Χ	△	Χ	Χ	△	△	Χ	○
Eddy/Eddy	○	Χ	○	△	○	△	△	Χ	○	△	○	△	△	Χ
S-type/Eddy	△	△	○	△	△	○	△	△	△	Χ	△	Χ	△	○
Left Loop/S-type	Χ	△	Χ	Χ	Χ	△	○	Χ	Χ	△	△	Χ	Χ	○
S-type/S-type	△	Χ	△	△	△	△	△	△	△	Χ	Χ	△	△	○
Arch/Balloon	△	△	○	Χ	○	○	Χ	Χ	△	○	○	Χ	△	○
S-type/balloon	△	Χ	Χ	Χ	○	Χ	Χ	△	△	Χ	○	○	△	Χ
Balloon/Balloon	Χ	○	Χ	Χ	○	Χ	Χ	Χ	Χ	Χ	△	Χ	Χ	Χ

△ = within the interval; Χ = left of the interval (less than 3.6169); ○ = right of the interval (more than 3.8215).

**Table 4:** Fingerprint types number summary and proportions.

Fingerprint Type	Amount	Fingerprint Type	Amount	Fingerprint Type	Amount
Left Loop/Right Loop	14	Left Loop/Whorl	2	S-type/Eddy	1
Whorl/Right Loop	1	Whorl/Whorl	5	Left Loop/S-type	1
Eddy/Right Loop	2	Eddy/Whorl	2	S-type/S-type	6
Left Loop/Tent	1	Eddy/Eddy	9	Balloon/Balloon	1

**Table 5:** Relationship between fingerprint type and personality traits.

	Socially Harmonious Method of Operation	Sentimentalism	Liveliness	Concern for Others’ Well-Being.	Strong Self-Esteem	Impatience	Impulsiveness	Positive Attitude	Strong Sense of Responsibility	Slow Method of Operation	Passivity	Focused Attention	Strong Leadership Ability	Good Money Concept
Left Loop/Right Loop	△	△	△	△	△	△	△	○	△	△	△	△	△	Χ
Whorl/Right Loop	Χ	Χ	○	Χ	Χ	△	○	Χ	○	Χ	△	Χ	Χ	○
Eddy/Right Loop	○	△	△	○	Χ	△	○	Χ	Χ	△	○	△	Χ	○
Left Loop/Tent	△	○	○	Χ	○	△	○	△	○	△	Χ	Χ	△	○
Left Loop/Whorl	○	△	△	Χ	Χ	Χ	○	○	△	Χ	Χ	Χ	○	Χ
Whorl/Whorl	△	○	△	△	○	○	○	○	△	△	Χ	Χ	Χ	△
Vortex/Whorl	Χ	△	Χ	Χ	○	△	△	○	Χ	△	○	△	△	○
Vortex/Vortex	○	Χ	○	△	○	Χ	△	Χ	○	△	○	△	△	△
S-type/Eddy	○	Χ	△	○	○	△	△	△	○	△	Χ	○	△	○
Left Loop/S-type	○	○	△	Χ	Χ	Χ	Χ	Χ	Χ	Χ	○	○	Χ	Χ
S-type/S-type	△	Χ	Χ	△	△	△	△	△	△	△	Χ	△	△	○
Balloon/Balloon	Χ	○	△	○	○	△	○	△	Χ	○	Χ	Χ	Χ	○

Discrepancy between pre-test and post-test shown in the highlighted cell, △ = within the interval; Χ = left of the interval; ○ = right of the interval.

**Table 6:** Results of the accuracy survey.

Mean	7.126801153	Minimum	1
Standard error	0.116532499	Maximum	10
Standard deviation	2.170759935	Total	2473
Variance	4.712198697	Number of results	351
Mode	8	Median	8
